# Defining patient communication needs during hospitalization to improve patient experience and health literacy

**DOI:** 10.1186/s12913-020-4991-3

**Published:** 2020-02-21

**Authors:** Guillem Marca-Frances, Joan Frigola-Reig, Jesica A. Menéndez-Signorini, Marc Compte-Pujol, Eulàlia Massana-Morera

**Affiliations:** Universitat de Vic - Universitat Central de Catalunya, Vic, Spain

**Keywords:** Health communication, Hospitalization, Health literacy

## Abstract

**Background:**

In order to play an active role in their health care, patients need information and motivation. Current delivery systems limit patients’ involvement because they do not routinely provide them with enough details of their own clinical results, conditions and other important clinical data. The purpose of this study was to identify, from the perspective of patients, which topics matter the most, who should be communicating them, and when and how should they be provided.

**Methods:**

We conducted a qualitative, phenomenological study analysing the content of subjective experiences, feelings and behaviours. We organized two focus groups with 13 participants and 15 in-depth interviews. Transcripts of the focus groups and interviews were checked for accuracy and then entered into Atlas ti™ v7.5.13 qualitative software. Two independent researchers performed a qualitative inductive content analysis to classify the data in two levels: themes and categories.

**Results:**

The qualitative analysis provided 377 units of meaning synthesized into 22 categories and six themes: hospitalization procedure, Health Literacy relating to the patient’s condition, information content, satisfaction, professional-patient relationship, and patient proactivity. Patients described which information they wished for, when they needed it, and who would provide it, usually related to actions such as admission, discharge or diagnostic tests. Oral information was more difficult to comprehend than the written kind, as patients can check written information several times if needed. Nurses were the most available professionals, and patients found easier to relate to them and ask them questions. Moreover, patients identified physicians as those professionals responsible for providing clinical information.

**Conclusions:**

Our results showed that patients suffered from poor Health Literacy regarding their personal condition, as they were unable to describe the symptoms, the type of tests being performed or their results, and some of them also had difficulties in naming the specific disease or comorbidities they had. During the hospitalization process, patients were in good shape to come with doubts and actively asked for more information. Healthcare organizations and professionals were offered the chance to ensure the correct communication and comprehension to their patients.

## Background

Positive patient experience is becoming more important for hospitals, and positive experiences have been closely linked to high employee engagement and quality of care [1]. At the same time, the ambition of the healthcare market is to transition towards a more value-based approach including patient-centred care models [2, 3]. In 2017, The Beryl Institute asked hospitals about the current overall focus and priorities for healthcare organizations. Its report pointed out that in 82% of cases the top priority was patient experience, which encompassed quality, safety and service [4]. It has been stated that patient experience, clinical effectiveness and patient safety are three dimensions of quality that should be explored as a whole group.

Patients’ perceptions about the quality of health care they receive depend to a large extent on the quality of their interactions with their doctor and the team of health professionals [5]. Additionally, it has been demonstrated that when doctors communicate better, the coordination of care and compliance with treatment also improves. Effective doctor-patient communication promotes compliance in medication and a more active self-management of long-term chronic conditions [6].

At the same time, improving nurses’ work environments, including staffing levels, may improve patient experience and quality of care [7]. It is known that nurses spend a lot of time with patients [8] and their communicative and social capabilities are important. In this sense, a Dutch study found that nurses can serve as spokespersons for patients who are often in vulnerable positions, and that they are also easily accessible and can act as a link between the patient and other professionals [9].

There is evidence that doctors’ and nurses’ communication skills are key influencers to patient experience and additionally, these competences are also closely related to hospital quality and effectiveness. In this context, understanding the needs of patients in terms of enabling access to the information that matters to them can provide key inputs for hospitals to become more active and consistent in standardizing their communication tactics (Laurence, Health Affairs) as an organization. Moreover, it can trigger an improvement strategy within its clinical staff by engaging them in training and education in communication and other social capabilities. A recent literature review concluded that patient engagement had a positive impact on health outcomes, medication adherence, and rates of hospital admission, which ultimately means a reduction in consumption of services and therefore, reducing costs and healthcare expenditure [10]. That is to say, in order to play an active role in their health care, patients need information that they understand, and motivation. Current delivery systems limit patients’ involvement by failing to routinely provide people with details of their own clinical results and conditions and other important clinical data. Currently, some delivery systems limit patients’ involvement because they do not routinely provide them with enough details of their own clinical results, conditions, and other important clinical data (3). Clinical professionals operate according to clinical guidelines, but these do not include when specific clinical information should be provided to patients in order to ensure patient engagement and motivation.

## Methods

### Aim of the study

The aim of this study was to identify, from the perspective of patients, who are the people that matter most, who should be communicating information to them and when and how this information should be provided.

### Research design and setting of the study

We conducted a qualitative, phenomenological study. We analysed the content of the subjective experiences, feelings and behaviours reported by patients concerning their recent hospitalizations, which were due to an exacerbation of their chronic conditions. The setting of the study was the University Hospital of Vic, located in the city of Vic. This is s referral hospital in a rural area in northeast Spain, providing care for 157,000 citizens. It admits around 5000 inpatients yearly, with 67% coming from the Emergency Room [11].

### Participants and data collection

Patients considered eligible for this study were those who living in their family homes and who were admitted to hospital after an exacerbation of their chronic condition. The chronic conditions were Chronic Obstructive Pulmonary Disease (COPD) and Chronic Heart Failure (CHF); additionally, we also considered Diabetes Mellitus type II as comorbidity when its relevance to the hospitalization episode was identified according to medical criteria. We excluded patients who suffered from any psychological or mental disease that could hinder the comprehension of any content, and those who were in an advanced stage, in which a fatal outcome within a period of less than 3 months was expected or in which palliative care had been requested. Institutionalized patients were also excluded from the study. The eligibility to participate in the study was assessed when patients were admitted to the hospital ward coming from the emergency room.

We conducted two focus groups including 13 participants in total: 5 relatives and 8 patients. In order to ensure participation, both focus groups were performed on the same day as a follow-up visit after their discharge, usually no later than 2 weeks, in the outpatient clinic of the hospital. In addition, we conducted interviews with 17 patients during their hospitalization (See additional file 1).

The focus of the study was the period established between admission to the Emergency Room and discharge from hospital. Patients were asked to participate in the study and were informed of the content and purpose of the study, before signing a consent form. Regarding the focus group, participants signed a consent form just before the meeting was performed, and they received all the information about the study once more in order to ensure their understanding. The in-depth interviews and focus groups were performed between April and June 2018. The language used was Catalan, which was the native language of all participants. The questions and situations shared with the participants of both the interviews and the focus group were proposed and validated by the clinicians working on the respiratory unit of the hospital. Additionally, we asked XPABarcelona [12], an independent group of experts in patient experience, to also provide their input on the questions and situations presented to the patients, to which they shared their opinions and own experiences.

The contributions made during the sessions were recorded with a digital recording device and then transcribed literally. The average duration of the focus groups was around 90 min, and 45 min for the interviews. The average length of the transcripts was 18 pages for focus groups and 5 for each interview, giving a total of 83 pages of analysed information.

In order to facilitate communication and establish a climate of trust, the goals of the study were explained at the beginning of each session and interview, guaranteeing the anonymity of the participants and the confidentiality of the data, and offering the possibility to stop and leave at any time of the session or interview without needing to give a reason.

### Ethical considerations

The Ethics and Research Committee from the Osona Foundation for Health Research and Education (FORES) approved the study protocol. All participants received written and verbal information about the aim and content of the study. Study participation was voluntary. Data were analysed in an anonymous way and the results were non-traceable to individual participants.

### Data analysis

The analysis of the data followed the steps of conventional qualitative inductive content analysis [13, 14]. Transcripts of the focus groups and interviews were checked for accuracy and then entered into Atlas ti™ v7.5.13 qualitative software. We conducted an initial analysis of the transcriptions so that we had an overall idea of its content in terms of relevance of the topics raised by patients and relatives and the approach to the topics we considered relevant. In a second reading, a segmentation of the data was carried out. To do this, we organized fragments that reflected the same idea (fragments of the text with semantic meaning) into units of meaning.

We used the method of constant comparisons, in which two researchers independently read the transcribed contributions of the participants in the focus groups and interviews, trying to find units of meaning that allowed the indexation of the fragments that described similar ideas. These theoretical segments were compared among researchers and once a consensus was achieved the next step was to individually code all the transcripts. The two investigators analysed their results, reconciled differences and reached a 100% consensus of their application of the coding for the information to be entered in the database. The data from the 2 focus groups and 17 interviews was divided into the following progressive levels of segmenting and structuring of the information:

- Level 1: segmentation and identification of units of meaning, into descriptive categories.
- Level 2: construction of a system of themes, including several units of meaning or categories.

On this data, the two researchers also analysed whether they transmit a positive, negative or neutral assessment. The researchers classified the messages according to the assessment patients added to their messages, in case positive or negative was not possible to identify, the message was classified as neutral.

## Results

We performed two focus groups of 6 and 7 participants, of whom 2 patients came with a caregiver (one per different group and considered them as a participant aside from the patient), and 15 interviews with hospital inpatients. Participants ranged in age from 54 to 86 (μ = 73.5; SD = 7.8). All respondents were Caucasian (91%, *n* = 31) and 70.8% were male (*n* = 17).

### Themes

The qualitative analysis of focus groups and interviews provided 377 units of meaning synthesized into 22 categories and 6 themes (Table 1). Participant gender, age, and study ID number are provided in brackets with corresponding quotes.

**Table 1:** Themes and categories with code frequency

	Interviews	Focus Groups	Total
**Hospitalization procedure**	**102**	**35**	**137**
Emergency Room admission	22	7	29
Hospital Stay	8	4	12
Hospital discharge	46	8	54
in-hospital procedures	26	16	42
**Health literacy around patient’s condition**	**118**	**51**	**169**
Background	23	20	43
Diagnose	38	14	52
Disease improvement	13	4	17
Knowledge about symptoms	13	4	17
Poor knowledge	31	9	40
**Information content**	**163**	**41**	**204**
Information on the admission	37	11	48
Information provided by physician	53	16	69
Understanding of the information	63	11	74
Lack of information	10	3	13
**Satisfaction**	**85**	**37**	**122**
Satisfaction with healthcare professionals	28	7	35
Satisfaction with services	22	3	25
Complaints	12	20	32
Discomfort	23	7	30
**Professional-patient relationship**	**48**	**17**	**65**
Trust with the professional	5	4	9
Shared decision making	6	0	6
Professional care	26	11	37
**Patient proactivity**	**25**	**10**	**35**
Family context	6	3	9
Behaviour & attitude	19	7	26
**Total**	**530**	**189**	**719**

#### Hospitalization procedure

The focus of this theme was to highlight the content regarding the whole procedure of hospitalization, from admission to the emergency room (ER) until discharge from hospital. This theme had four categories: emergency room admission, hospital stay, discharge, and in-hospital procedures.

All patients knew that the entry point to the hospital was through the ER. Due to their chronic condition, patients were often in the ER and they knew the dynamics of the process. However, being in the ER created anxiety, especially because patients felt they did not get information at the right time and pace: “I get nervous when I go to the ER, time runs, I know they are doing their job, but I do not understand what they are doing” (M,80,1). “They do some tests on me, but they keep the results” (D,72,15) “Everything was slow, and they do not give information, I just knew that I felt shortness of breath”.

During hospitalization, the nurse was the closest professional to the patient. However, nurses tended to avoid conversations regarding the disease, its management or treatment and they chose more generic or vague topics to engage with patients: “nurses do not give much information, they do not speak about the disease, but they encouraged me” (M,77,2). “When I asked about the results of a test, the nurse told me that the physician would eventually talk to me later” (M,66,10). These sentences confirm that nurses were the closest clinical professionals to patients and that patients did not hesitate to ask them for information.

When being discharged, participants noted that the doctor was the one giving the news and explaining the treatment details. However, participants stated that they were not aware of their physician’s name, they could not evaluate if they were good enough to be discharged and that they did not understand the treatment instructions they had to follow at home. “I know the physician gave me the discharge order, but I do not know his name” (M,65,1), “they gave me the discharge order because they say I am better now” (M,80,3), “I do not really understand the discharge instructions, but I do not mind, I will go through them at home” (M,65,1). With these examples, we can also note that patients are given a medical report when being discharged, and that they rely on the possibility of going through the instructions as many times as they need when they arrive home.

#### Health literacy around the patient’s condition

This theme includes 5 categories: background, diagnosis, improvement in the disease, knowledge of symptoms, and poor knowledge. Specifically, patients were asked to talk about their knowledge and understanding of what happened to them regarding their current condition. In general, patients tended to literally describe their experience and they frequently went back to the moment in time when they were first aware of their chronic condition. In this sense, patients commented on the first times they felt shortness of breath, and those who smoke commented on when they were told to stop smoking and actually quitted: “I was diagnosed two years ago. I started to get tired and they told me you have to stop smoking, and I said, I’ll quit” (D,54,7). Additionally, they commonly related the moment of diagnosis to a hospitalization episode: “It was in December 2016, I was in the hospital for one month” (M,74,5). Another moment of being aware of the diagnosis is related to an important change in their lives, such as the need to stop working, “the worst was I had to stop working”.

The knowledge of symptoms is poor and patients tend to mix conditions, such as respiratory and cardiac, acute and chronic, etc. “It felt like having 25 kgs on my chest, I could not breathe [...]” (M,80,8); “I felt my hands and my head burn, I felt strange and I thought: “if I have a fever, I have an infection”” (M,83,11). When patients were specifically asked about the name of the condition or the results of the tests performed on them, they cannot name any specific disease and they cannot describe the results of the tests either: “They did not mention a disease that was bad and all that [...] they have never told me that I have anything, but they tell me that I have high blood pressure, I get a cold almost every year [...] in the past three years I have been in the hospital three times for the same thing” (D,86,6). Patients felt they are not informed and their attitude towards their situation is passive and conformist: “They do not give me any information [...] when the doctor comes, then yes [...] but they do not explain much” (M,83,9); “They did not give me information [...] they have explained that I am good enough to go and that my heart is fine” (M,80,8). Participants did not mention not being able to properly understand when doctors or nurses tried to explain matters related to their chronic condition, or the acute episode that caused their hospitalization.

#### Information content

This theme includes 4 categories: information on admission, information provided by the physician, understanding of the information, and lack of information. This theme summarizes all the feelings and behaviours from the patient that specifically deal with information.

Patients were asked if they were informed about the decision and the reason to admit them to hospital in the ER. All patients stated that they were informed about the admission and the reason why, either in the ER or on the hospital ward: “Downstairs (ER) I was very well informed that I had an infection and I had to stay” (D,70,3). However, if patients have respiratory symptoms, they can be confused and some participants mentioned having difficulties in remembering the information they were told: “I do not know if we talked about it, but I already knew it, because I felt sick” (M,83,9). Participants agree on the physician being the person providing the diagnosis, and some patients explain that they got the information in the hospital, as opposed to not being informed while being treated in Primary care: “The doctor gave me the information, in detail” (M,80,2); “Here [ward] the doctor told me I had COPD. The family doctor did not tell me that I had COPD, but he told me I had to stop smoking” (D,54,7). Additionally, some patients mention that they look for additional explanations on the Internet, knowing that not everything they find is to be trusted: “it’s pretty clear, what happens is that I may be looking for a lot of information myself. [...] On the Internet, besides I know that not everything I find on the Internet can be trusted [...]” (M,74,5).

#### Satisfaction

This theme includes 4 categories, which are: satisfaction with healthcare professionals, satisfaction with services, complaints, and discomfort. When being asked about this theme, participants of the study did not give negative messages and in fact, none of them had any complaints about their hospital stay. Some of them mentioned that when being curious about certain matters, they were not fully satisfied with the level of detail of the explanations they were given, and in case of asking several professionals, they thought that the information provided was not consistent: “If I ask, for example [...]. Why have you put me six different intravenous routes?” (M,74,5), “I am afraid because one tells me one thing and somebody else tells me another [...] and I feel insecure” (M,77,2). On the other hand, patients feel they were in good hands and they fully rely on professionals and the healthcare system: ‘Yes, I swear to thank you what you did today with me, because I was quite scared and you gave me security’ (M,80,1); The hospital treatment, the doctors and everything, truly wonderful, I do not have anything to say at all” (M,80,2).

Regarding the services, some participants recall that they were told about the existence of an alarm button by the bedside and that they could always press it and somebody would respond: “They [professionals] all say, “any time you need someone, you just have to ring the bell” (M,66,10).

#### Professional-patient relationship

This theme includes 3 categories, which are: trust in healthcare professionals, shared decision making, and professional care. Regarding the first category, trust in healthcare professionals, the responses of patients are unanimous and in all cases the reply is of absolute trust: “If you see it like that, I do not understand anything, but you are the expert” (M,66,10); “I did what I was told and that is all, yes!” (M,77,2); “I never doubt the professional [...] because he tells the truth, at least in my case he always did to me” (M,80,2). Additionally, patients’ response to the improvement of their wellbeing during the hospitalization period if they had actively participated in taking decisions about themselves was negative: “I would say no” (F,69,4).

Participants described professional care according to what they could see by themselves, as spectators of what happened around them. At this point, there were several comments describing the frequency of the visits made by the physician, such as “the doctor came every day”, the physician being the person explaining everything, and also how physicians and nurses interacted and their respective roles: “Nurses are the ones doing it [...] following the doctor’s orders [...] the doctor comes and tells them “now here go down or go up or do it [...] “Then the doctor says “we will wait two more days because this is still not fully recovered “Or “we could wait until tomorrow, or you can leave today “, but it will be the doctor who will come with the results they have of the whole process” (M,76,12).

#### Patient proactivity

This theme includes two categories, which are: family context, and behaviour & attitude. Regarding the first one, participants’ comments were focused on the role of caregivers and, more specifically, in taking care of medical prescriptions. In most cases, the caregivers were relatives, and they comment on always being aware of any changes in the name or number of drugs, why the drugs were prescribed, the preparation of the dosage. Therefore, caregivers were in charge of ensuring drug treatment compliance: “[relative] I am the one in charge of the medication, it is me. I am, you [patient] can look at it but I put the medications “[...] [patient]: Now I do not take care of anything, isn’t it? As of last admission to the hospital she [relative] does everything” (F,70,3). There were a few comments in the category of behaviour & attitude where the participant provided additional information to an affirmative or negative answer when being asked. At this point, we selected one response that defined a quite common reasoning among patients, given the chronicity of their conditions and the fact that all of them were frequent users of the healthcare system: “[Patient recommending another patient] I recommend that when you feel drowned you should ask your doctor to use the machine called CPAP [...] In my case I was at 87, 88 and even down to 70 [...] of oxygen saturation” (F,65,4).

In Table 2 we show the classification of the different mentions into positive, negative and neutral.

**Table 2:** Themes and categories with code frequency and type of response

	Interviews				Focus Groups				Total			
	*Positive*	*Neutral*	*Negative*		*Positive*	*Neutral*	*Negative*		*Positive*	*Neutral*	*Negative*	
**Hospitalization procedure**				**102**				**35**				**137**
Emergency Room admission	0 (0%)	14 (63.6%)	8 (36.4%)	22	0 (0%)	4 (57.1%)	3 (42.9%)	7	0 (0%)	18 (62.1%)	11 (37.9%)	29
Hospital Stay	0 (0%)	8 (100%)	0 (0%)	8	1 (25%)	3 (75%)	0 (0%)	4	1 (8.3%)	11 (91.7%)	0 (0%)	12
Hospital discharge	3 (6.5%)	42 (91.3%)	1 (2.2%)	46	0 (0%)	10 (100%)	0 (0%)	8	3 (5.6%)	50 (92.6%)	1 (1.9%)	54
in-hospital procedures	2 (7.7%)	21 (80.8%)	3 (11.5%)	26	1 (6.3%)	14 (87.5%)	1 (6.3%)	16	3 (7.1%)	35 (83.3%)	4 (9.5%)	42
**Health literacy around patient’s condition**				**118**				**51**				**169**
Background	0 (0%)	23 (100%)	0 (0%)	23	0 (0%)	20 (100%)	0 (0%)	20	0 (0%)	43 (100%)	0 (0%)	43
Diagnose	0 (0%)	37 (97.4%)	1 (2.6%)	38	0 (0%)	14 (100%)	0 (0%)	14	0 (0%)	51 (98.1%)	1 (1.9%)	52
Disease improvement	0 (0%)	12 (100%)	0 (0%)	13	1 (20%)	4 (80%)	0 (0%)	4	1 (5.9%)	16 (94.1%)	0 (0%)	17
Knowledge about symptoms	0 (0%)	13 (100%)	0 (0%)	13	0 (0%)	4 (100%)	0 (0%)	4	0 (0%)	17 (100%)	0 (0%)	17
Poor knowledge	0 (0%)	29 (96.7%)	1 (3.3%)	31	0 (0%)	8 (88.9%)	1 (11.1%)	9	0 (0%)	37 (94.9%)	2 (5.1%)	40
**Information content**				**163**				**41**				**204**
Information on the admission	4 (12.5%)	27 (84.4%)	1 (3.1%)	37	2 (20%)	8 (80%)	0 (0%)	11	6 (14.3%)	35 (83.3%)	1 (2.4%)	48
Information provided by physician	3 (5.7%)	49 (92.5%)	1 (1.9%)	53	2 (12.5%)	12 (75%)	2 (12.5%)	16	5 (7.2%)	61 (88.4%)	3 (4.3%)	69
Understanding of the information	6 (9.5%)	53 (84.1%)	4 (6.3%)	63	1 (9.1%)	9 (81.1%)	1 (9.1%)	11	7 (9.5%)	62 (83.8%)	5 (6.8%)	74
Lack of information	0 (0%)	8 (100%)	2 (20%)	10	0 (0%)	3 (100%)	0 (0%)	3	0 (0%)	11 (84.6%)	2 (14.5%)	13
**Satisfaction**				**85**				**37**				**122**
Satisfaction with healthcare professionals	25 (89.3%)	0 (0%)	3 (10.7%)	28	5 (71.4%)	0 (0%)	3 (28.6%)	7	30 (85.7%)	0 (0%)	5 (14.3%)	35
Satisfaction with services	19 (86.4%)	1 (4.5%)	2 (9.1%)	22	2 (66.7%)	0 (0%)	1 (33.3%)	3	21 (84%)	1 (4%)	3 (12%)	25
Complaints	0 (0%)	0 (0%)	12 (100%)	12	0 (0%)	0 (0%)	20 (100%)	20	0 (0%)	0 (0%)	32 (100%)	32
Discomfort	0 (0%)	21 (91.3%)	2 (8.7%)	23	0 (0%)	5 (71.4%)	2 (28.6%)	7	0 (0%)	26 (86.7%)	4 (13.3%)	30
**Professional-patient relationship**				**48**				**17**				**65**
Trust with the professional	0 (0%)	5 (100%)	0 (0%)	5	1 (25%)	3 (75%)	0 (0%)	4	1 (11.1%)	8 (88.9%)	0 (0%)	9
Shared decision making	0 (0%)	6 (100%)	0 (0%)	6	0 (0%)	0 (0%)	0 (0%)	0	0 (0%)	6 (100%)	0 (0%)	6
Professional care	0 (0%)	26 (100%)	0 (0%)	26	0 (0%)	11 (100%)	0 (0%)	11	0 (0%)	37 (100%)	0 (0%)	37
**Patient proactivity**				**25**				**10**				**35**
Family context	1 (16.7%)	5 (83.3%)	0 (0%)	6	0 (0%)	3 (100%)	0 (0%)	3	1 (11.1%)	8 (88.9%)	0 (0%)	9
Behaviour & attitude	1 (5.3%)	18 (94.7%)	0 (0%)	19	1 (14.3%)	6 (85.7%)	0 (0%)	7	2 (7.7%)	24 (92.3%)	0 (0%)	26

The types of response obtained show that, except for the elements directly linked to satisfaction, most of the topics present neutral answers. The only exception is when patients are in the emergency room, a situation which generates more anxiety, turning their response into a more negative opinion.

## Discussion

Our research aimed to identify which topics mattered most to patients suffering from chronic conditions and who, when and how these topics should be communicated. This was achieved by undertaking a qualitative analysis of the experiences described by patients during a recent hospitalization or after discharge from hospital. The themes that were mentioned more frequently in the interviews and focus groups were: Information content, Health Literacy (HL) around patients’ conditions and hospitalization procedure. Categories mentioned more frequently during hospitalization appear to be those related to the in-hospital procedures and discharge which are closely linked to actions that patients can easily identify: the tests being performed and the disclosure of the results and discharge. However, when being asked, patients are unable to share details regarding these categories because they do not understand what the test entails, which results they will provide, or any further consequences arising from the results. They all knew or were told that any clinical information was disclosed by physicians and through oral communication. Patients recognized the convenience of having everything written in a discharge report, which they can repeatedly consult it in case of doubts, while alone. This is in fact an important element as it demonstrates the usefulness of providing written schemes and information on any topic, in this case discharge reports. Written information is not provided while in the ER, and this lack of information triggers stress in patients, nor is it provided during the hospitalization process. Moreover, the hospitalization period offers a good opportunity to educate patients as they can actively ask nurses if they have any queries who, according to our results, appear to be the professionals who were frequently asked for information by patients [8].

Considering the aspects that require patients to take action on their self-care while at home, there is complete understanding about the need of drug treatment and even caregivers take active responsibility in ensuring compliance. However, patients did not mention any specific recommendations on lifestyle, which are also important in preventing exacerbations and promoting a full recovery. In this area, patients only mentioned the need to stop smoking, specially linked to the moment of diagnosis. The content analysis of the discharge reports was not under the scope of this study, but given the importance of written information in discharge reports, it is highly recommended that they deal with frequent comorbidities and include lifestyle recommendations in terms of physical exercise, nutrition, respiratory rehabilitation, etc. When back home, the management of the patient’s condition – a condition that takes place 24 h a day, 7 days a week, requires knowledge and active participation from the patient [15].

When we look at the results, we can clarify about who should provide the information and how. Patients are aware that physicians are the ones responsible for the disclosure of new and important information. However, patients have more access to nurses and they frequently engage them in conversations. In this sense, nurses seem the most accessible professionals for doubts and they should be encouraged to respond and be specifically trained to improve their communication skills to patients with poor Health Literacy. In relation to how, verbal communication appears to be the most convenient for professionals, but it is not appropriate as a stand-alone communication action to ensure patient understanding. This leads us to the conclusion of the appropriateness of providing written information while in the ER and on the hospital ward, offering the possibility not only to learn more about the current situation, but also to have a more comprehensive understanding about the one or several chronic conditions they suffer. This written information could be co-created by patients and healthcare professionals [16, 17].

When we look at the comments made by patients concerning when they should be provided with this information, we identified some key moments where information is either related to actions such as: admission, results from tests, discharge, etc. or related to doubts about the information they were given. If the information provided in the first place is given in the right way, not only orally but also written and in plain language, any doubts might be fewer in number and easy to respond to by referring patients to the primary source. It is possible that while physicians will lead the initial explanations and information disclosure, nurses might be the most frequent recipients of patients’ questions concerning the doubts [7, 8].

Our results showed that patients had little understanding of their personal condition, as they were unable to describe their symptoms, the type of tests being performed or their results and some of them had difficulties in naming their specific disease or comorbidities. The definition of Health Literacy is ‘Degree to which individuals have the capacity to obtain, process, and understand basic health information and services needed to make appropriate health decisions’ [18]. Therefore HL is a key element if we want to promote healthy and active ageing. Previous studies have shown that 58.3% of the general Spanish population had inadequate or problematic HL, and that having more than one long-term illness, having visited the doctor 6 times or more in the past year, having lower education, being 66 years old or older was associated to a higher percentages of inadequate or problematic HL [19].

As a study limitation, these results cannot be applied to the whole population, but it is possible that our findings are relevant to Spanish citizens living in rural areas, suffering from at least one chronic condition and being older than 65 years old. However, Spain presents a high percentage of population with inadequate HL who could benefit from the conclusions and derived action points in terms of improving health communication efficiency and patients’ knowledge. Another limitation to this study is that the goal of any communication is patient activation, however, the goals of this study are limited to describe patient experiences and report the findings. Because of the limited number of participants, we did not specifically describe the results coming from family caregivers that participated in the study. Due to the importance of the caregiver role in chronic patients, we recommend focusing on this area of study in future studies. Another limitation to this study is that it has a selection bias because it focuses on patients that have been admitted to the hospital, leaving out of the study those patients that are followed up by Primary Care.

## Conclusions

The results of our study show that healthcare organizations and professionals are offered the chance to ensure adequate communication and enhance HL improvement among the patients they treat. The population of our study matches most of the elements linked to poor HL such as being elderly, being frequent users of the healthcare system and suffering from more than one chronic condition.

Patients suffering from chronic conditions are often admitted to the ER and in some cases, hospitalized. We have seen that during the hospitalization process patients are able to identify doubts and actively ask for more information. These moments offer healthcare professionals the chance to provide personalized attention and information to support patients’ learning about their condition and comorbidities that will ultimately improve patient experience, enhance better treatment compliance and contribute to healthy ageing.

Finally, healthcare professionals should take responsibility for the opportunity that the hospitalization period provides to increase Health Literacy among frequent healthcare users [20]. It is therefore important to evaluate the impact of these action points in order to standardize communication and education initiatives within the clinical guidelines among hospitalized patients suffering from chronic conditions.

## Supplementary information

**Additional file 1.** Semi-structured in-depth interview guide.

## Data Availability

Data generated during the current study is not publicly available but upon reasonable request anonymous data could be provided if justified to the corresponding author.

## Abbreviations

CHF: Chronic Heart Failure
COPD: Chronic Obstructive Pulmonary Disease
ER: Emergency Room
HL: Health Literacy
