# Supplementary material for: Defining patient communication needs during hospitalization to improve patient experience and health literacy
Source: BMC Health Serv Res. 2020 Feb 21;20:131. doi: 10.1186/s12913-020-4991-3 (PMC7035644; doi:10.1186/s12913-020-4991-3)
Supplement: Supplementary file 1 — Additional file 1. Semi-structured in-depth interview guide. [file 12913_2020_4991_MOESM1_ESM.docx]

**SEMI-STRUCTURED IN-DEPTH INTERVIEW GUIDE**

1. Once you arrived at Hospital or Care Center, Do you remember the information received there? If so:

a. How would you define the Tone of the information explained?

b. Was the information clear and comprehensive?

c. Do you think the information was helpful enough to understand your situation as patient?

2. During the Hospital Admission Process, Who explained you those medical reasons needed for this decision? How was the decision making process?

a. Did you receive information about those medical reasons for hospital admittance?

b. How do you evaluate the information provided? How this information was providing?

c. Do you think that a proper medical information given prior to hospital admission process helps to understand the patient's situation?

3. Once the diagnosis and treatments are done, do you consider the clinical results were correctly explained by medical staff?

a. Who explained you this information?

b. When it comes to clinical diagnosis, do you think the terminology used is correct?

c. Did you participate on the decision making process about the procedure to follow once the clinical results are explained?

d. Are you satisfied on the personal attention given to you when you are receiving this clinical or medical information?

e. Have you had doubts regarding your diagnosis?

i. If so, were these doubts answered?

4. How would you evaluate your clinical evolution during the admission process?

a. During the medical stay, at what stage do you consider you have received the most amount of information?

b. During the medical stay, who was in charge of explain your current clinical situation at that time?

c. Was the information procedure always the same? Did you perceive any changes on the information procedure for certain medical conditions?

d. Do you think using more customized media channels and personalized communication methods can be helpful for patients to inform them about their medical diagnosis and/or treatments?

5. Regarding the discharge process, Do you think you received adequate and sufficient information about the decision and medical reasons?

a. Who communicated the information to you?

b. Were you able to take part on the decision making process?

c. In relation to discharge instructions, Do you consider the information provided was clear and understandable?

d. After the discharge, did you receive updated information about your medical situation?

6. Are you satisfied with the clinical and medical information received?

a. Do you think your participation should be taken into account in the discussion?

b. Do you consider the amount of information was correct or insufficient?

c. Did you have trouble understanding the information, due to its medical terminology?

d. In any case, did you use supportive techniques to make the explanations clearer?

7. What is your personal opinion about the hospital institution?

a. Regarding the information provide to patients, What kind of improvements do you think can be carried out to make the information more accessible?

b. Can you evaluate your relationship with the medical staff (doctors, nurses, administration staff, etc.) at the center as positive?

c. Do you think that patient’s participation on the decision making process is a good practice to improve communication during the hospital stay?
